# Supplementary material for: Negativeome characterization and decontamination in early-life virome studies
Source: Nat Commun. 2025 Jul 4;16:6190. doi: 10.1038/s41467-025-61478-7 (PMC12227732; doi:10.1038/s41467-025-61478-7)
Supplement: Supplementary file 1 — Supplementary information [file 41467_2025_61478_MOESM1_ESM.pdf]

Supplementary information: Negativeome characterization and decontamination in early-life virome studies

N. Kuzub et al.

Supplementary Figure 1: Study design and sample distribution per dataset.

Supplementary Figure 2: Characteristics of viral sequences used for species-level dereplication.

Supplementary Figure 3: Comparison of genomic and ecological features between biological samples and NCs.

Supplementary Figure 4: Comparison of virus and host-based taxonomical composition between biological samples and NCs.

Supplementary Figure 5: Comparison of the host-based vOTU aggregate composition between NCs and samples.

Supplementary Figure 6: Comparison of vOTU-level similarity index between NCs and samples versus between unrelated samples.

Supplementary Figure 7: Venn diagrams for the vOTUs detected in NCs and biological samples.

Supplementary Figure 8: Differential abundance of vOTUs shared between NCs and samples.

Supplementary Figure 9: vOTU and strain sharing between samples and external versus internal NCs.

Supplementary Figures

Sample type	Study	NC source / sample timepoint	N extracted	N deposited	N after read QC	N with non-zero richness	N sharing vOTUs to NCs
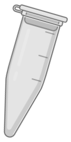 NCs	Garmaeva et al.	Buffer	4	1	1	1	1
	Liang et al.	Buffer; Tube; Diaper; MDNC	38	38	38	20	20
	Maqsood et al.	Buffer; Orsay	8	8	8	8	8
	Shah et al.	Buffer	8	8	8	8	8
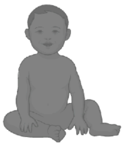 Infants	Garmaeva et al.	M1; M2; M3; M6; M12	129	86	86	86	72
	Liang et al.	M0; M1; M4; Y2-5	394	391	383	324	137
	Maqsood et al.	M0	56	51	51	51	42
	Shah et al.	M12	660	647	647	647	620
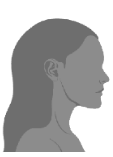 Mothers	Garmaeva et al.	Mtrim3; M0; M1; M2; M3	223	119	119	119	71
	Maqsood et al.	M0	28	27	27	27	2

Supplementary Figure 1. Study design and sample distribution per dataset. The third column provides the source of negative controls (NCs) for each study, while for biological samples, it lists the timepoints included per study. Timepoints are represented by the abbreviations M0, M1, M2, M3, M4, M6, and M12, corresponding to the infant's age in months at the time of sampling (M = month). Additionally, "Mtrim3" denotes samples collected during the mother's third trimester of pregnancy,

and "Y2-5" indicates samples collected from infants aged 2 to 5 years. Detailed information on the number of samples per timepoint for both infants and mothers can be found in Supplementary Data 1. Columns 4-8 provide further details: the number of samples that were extracted using viral-like particle enrichment protocol (N extracted); the number of samples deposited to the archives (N deposited); the number of samples with non-zero clean reads following read quality control (N after read QC); the number of samples where at least one vOTU was identified (N with non-zero richness); the number of samples that shared at least one vOTU with the NCs (N sharing vOTUs to NCs). Created in BioRender. Garmaeva, S. (2025) https://BioRender.com/ln81hl5.

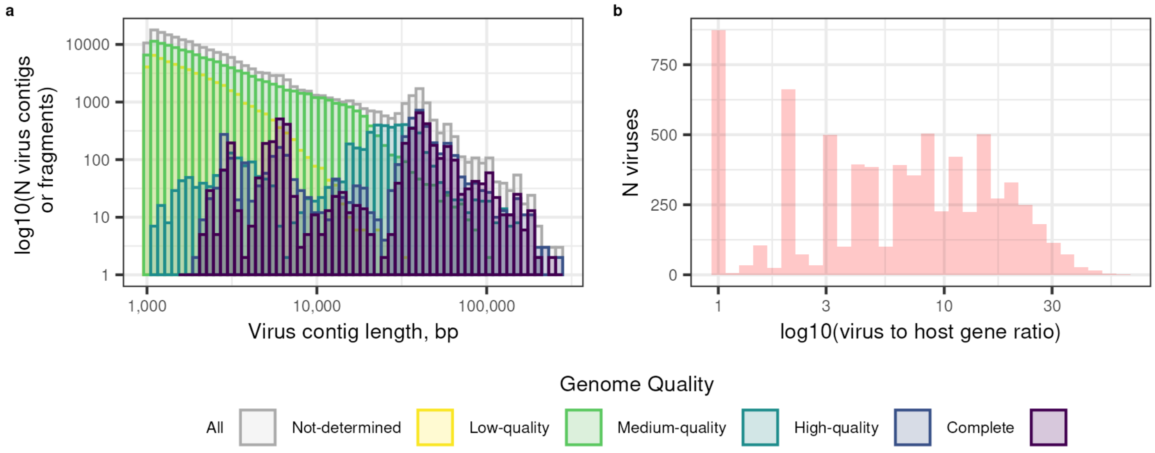

Supplementary Figure 2. Characteristics of viral sequences used for species-level dereplication. a. The length distribution of viral genomes and genome fractions colored by genome quality estimated by CheckV. The Y-axis is displayed on a logarithmic scale. **b.** Distribution of viral genomes and genome fractions based on the virus-to-host gene ratio. In **a-b,** the data presented reflects the virus genomes and genome fractions that fulfilled the following criteria: length > 1 kbp, a higher number of viral genes compared to host genes, and no plasmid sequences.

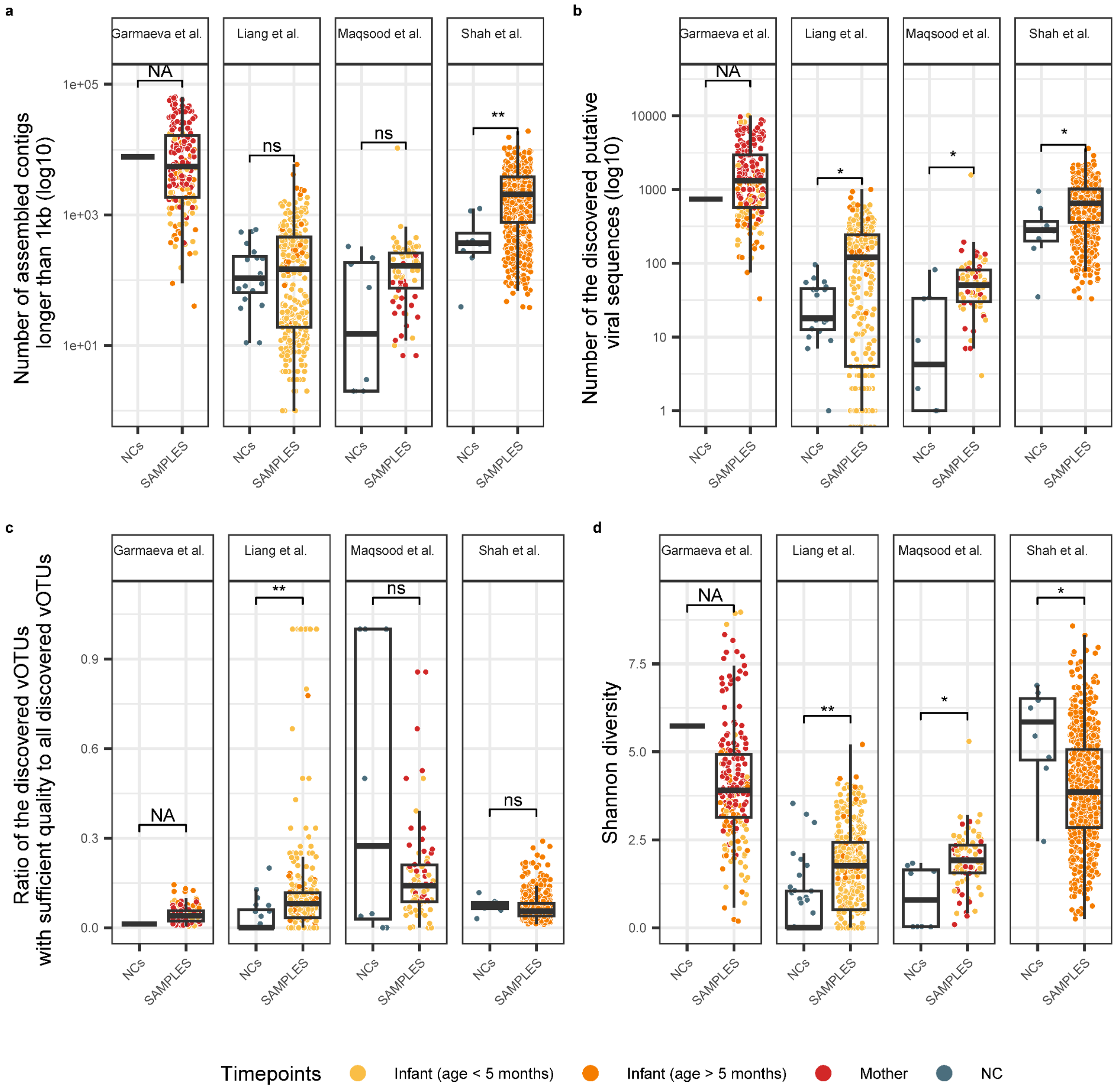

Supplementary Figure 3. Comparison of genomic and ecological features between biological samples and NCs. a. Number of assembled contigs longer than 1 kb in NCs compared to biological samples. **b.** Number of the discovered putative viral sequences in NCs vs samples. **c.** Ratio of the discovered putative viral sequences of sufficient quality to all putative viral sequences in NCs vs samples. Sufficient quality indicates viral sequences of at least 50% completeness as assessed by CheckV. **d.** Shannon diversity in NCs vs samples. In **a-b, d**, data are shown for 1,313 samples and 55 NCs, distributed by study as follows: Garmaeva et al. (NCs = 1, samples = 205); Liang et al. (NCs = 38, samples = 383); Maqsood et al. (NCs = 8, samples = 78); Shah et al. (NCs = 8, samples = 647). In **c**, data are shown for 1,254 samples and 37 NCs, distributed by study as follows: Garmaeva et al. (NCs

= 1, samples = 205); Liang et al. (NCs = 20, samples = 324); Maqsood et al. (NCs = 8, samples = 78); Shah et al. (NCs = 8, samples = 647). In **a-d,** each sample is a dot, and the dot color represents the age: infant samples (age < 5 months) in yellow, infant samples (age > 5 months) in orange, maternal samples in red, and NCs in dark blue. Boxplots visualize the median, hinges (25th and 75th percentiles), and whiskers extending up to 1.5 times the interquartile range from the hinges. In **a-c,** Y-axes are shown on the logarithmic scale. In **a**-**d** asterisks denote Benjamini-Hochberg-adjusted statistical significance values, *p-value < 0.05; **p-value < 0.01; ns=not significant. 'NA' is used when significance cannot be calculated because only a single NC is available. For details on the statistical test results, please see Supplementary Data 3–5 and 7.

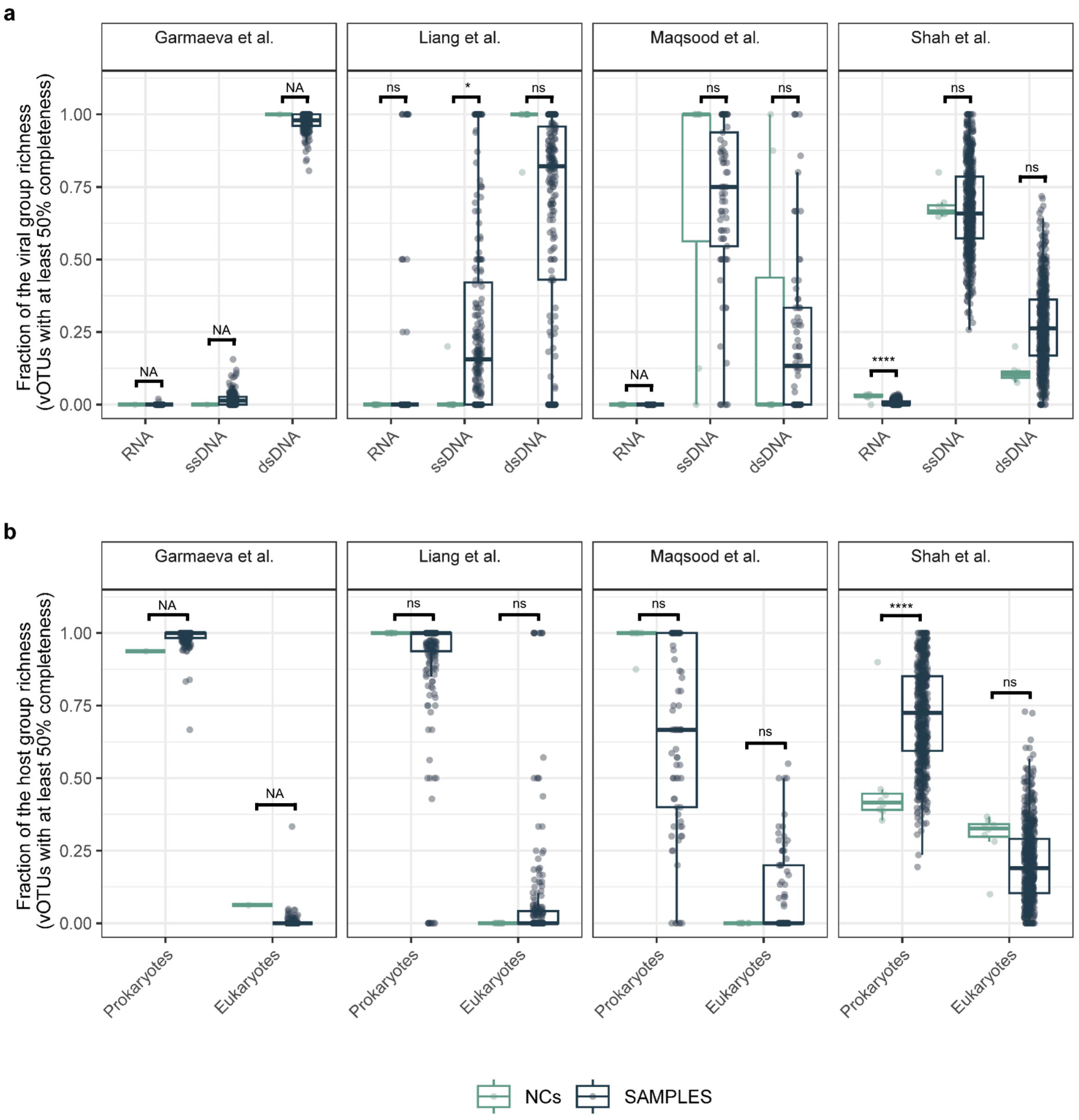

Supplementary Figure 4. Comparison of virus and host-based taxonomical composition between biological samples and NCs. a. Comparison of the distribution of vOTU richness proportions by nucleic acid type assigned via deduced virus taxonomy between NCs and samples. **b.** Comparison of the distribution of vOTU richness proportions by the assigned host domain (eukaryotic vs prokaryotic) between NCs and samples. In **a** and **b,** every dot is a sample and color indicates whether it is an NC (light blue), or a biological sample (dark blue). Data are shown for 1,254 samples and 37 NCs. Per study: Garmaeva et al. (NCs = 1, samples = 205); Liang et al. (NCs = 10, samples = 230); Maqsood et al. (NCs = 7, samples = 77); Shah et al. (NCs = 8, samples = 647). Boxplots visualize the median, hinges

(25th and 75th percentiles), and whiskers extending up to 1.5 times the interquartile range from the hinges. Asterisks denote Benjamini-Hochberg-adjusted statistical significance values, *p-value < 0.05; ****p-value < 0.0001; ns=not significant. 'NA' is used when significance cannot be calculated because only a single NC is available. For details on the statistical test results, please see Supplementary Data 9 and 11.

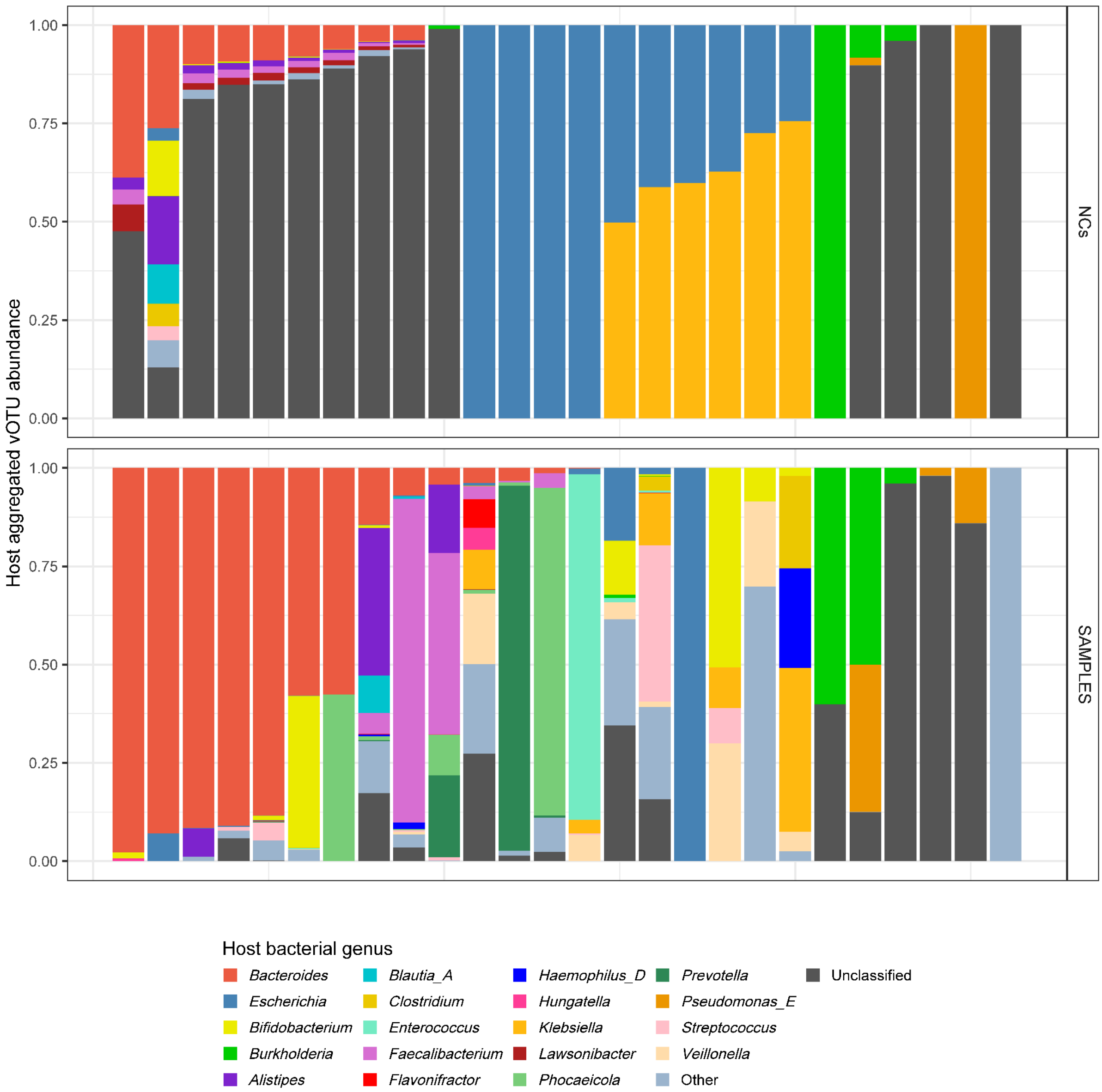

Supplementary Figure 5. Comparison of the host-based vOTU aggregate composition between NCs and samples. The plot displays the relative abundance of vOTUs with at least 50% genome completeness, aggregated by host composition. The upper panel represents all NCs with the non-zero richness of vOTUs with at least 50% genome completeness (n=26), while the lower panel includes the infant early-life samples, each randomly matched with an NC from the same cohort (n=26). The specific order and sample metadata are provided in Supplementary Data 25.

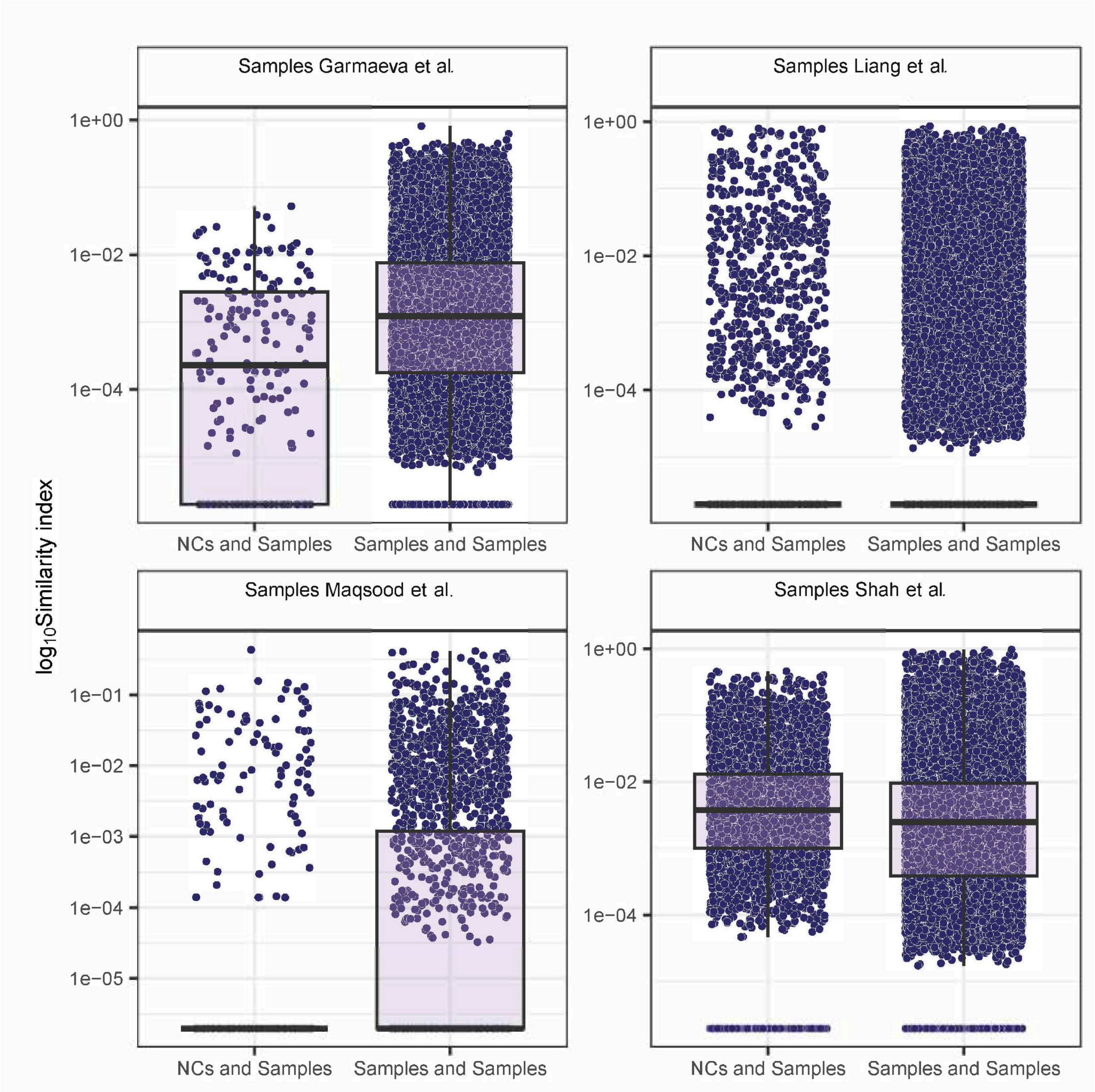

Supplementary Figure 6. Comparison of vOTU-level similarity index between NCs and samples versus between unrelated samples. In each panel, the left boxplot illustrates the similarity between NCs and samples, while the right boxplot shows the between-sample similarity within the same study. Between-sample similarities for samples of the same or related individuals are not shown. Each dot represents the log10-transformed similarity index between two samples, calculated as (1 - Bray-Curtis dissimilarity index). Number of depicted data points per study: Garmaeva et al. (NCs–Samples = 205, Samples–Samples = 20,244); Liang et al. (NCs–Samples = 6,480, Samples–Samples = 52,002); Maqsood et al. (NCs–Samples = 624, Samples–Samples = 2,930); Shah et al. (NCs–Samples = 5,176, Samples–Samples = 208,981). Boxplots visualize the median, hinges (25th

and 75th percentiles), and whiskers extending up to 1.5 times the interquartile range from the hinges. For details on the statistical test results, please see Supplementary Data 12.

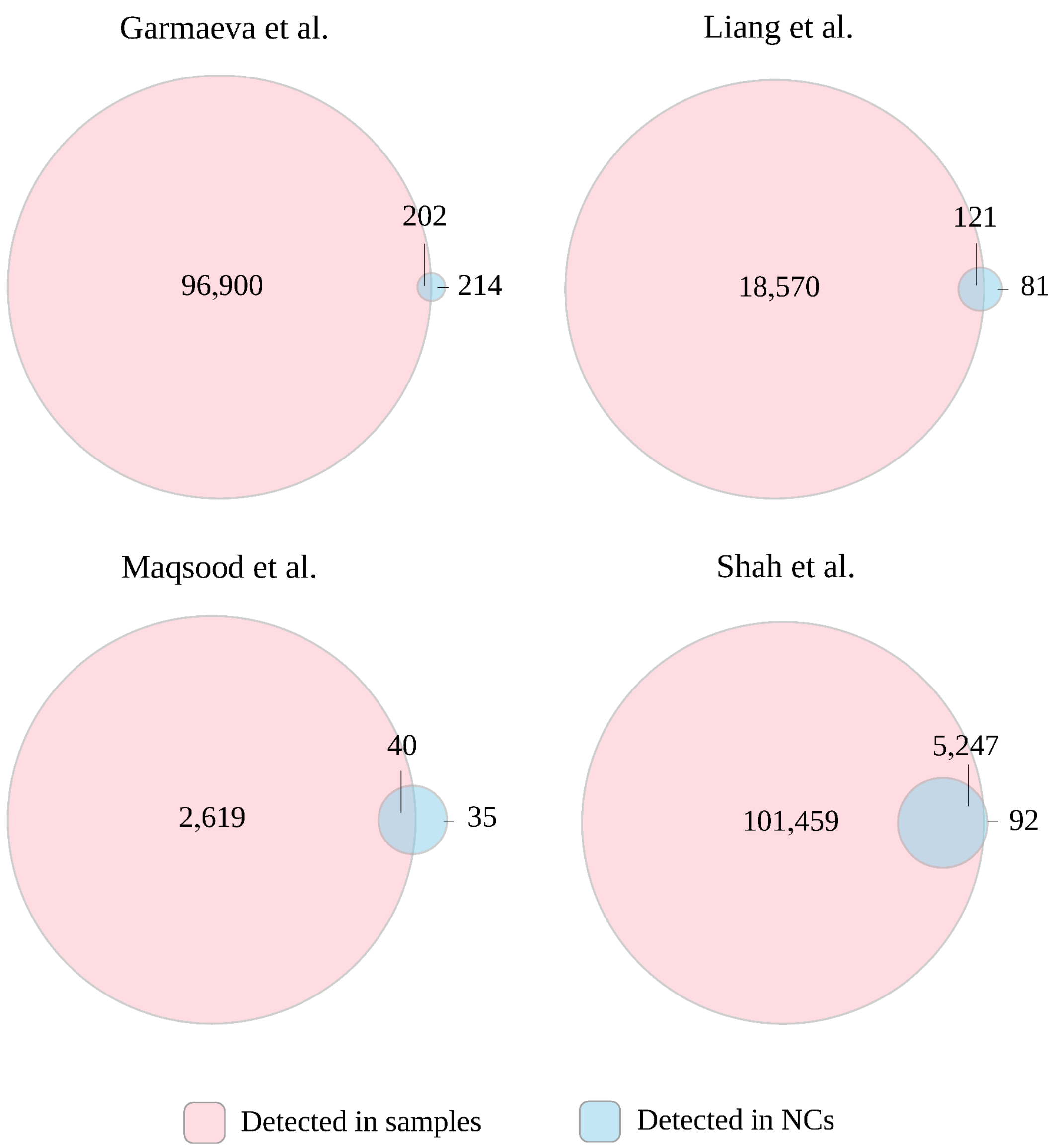

Supplementary Figure 7. Venn diagrams for the vOTUs detected in NCs and biological samples. vOTU sequences were detected in at least one sample or NC and in at least one of the four studies.

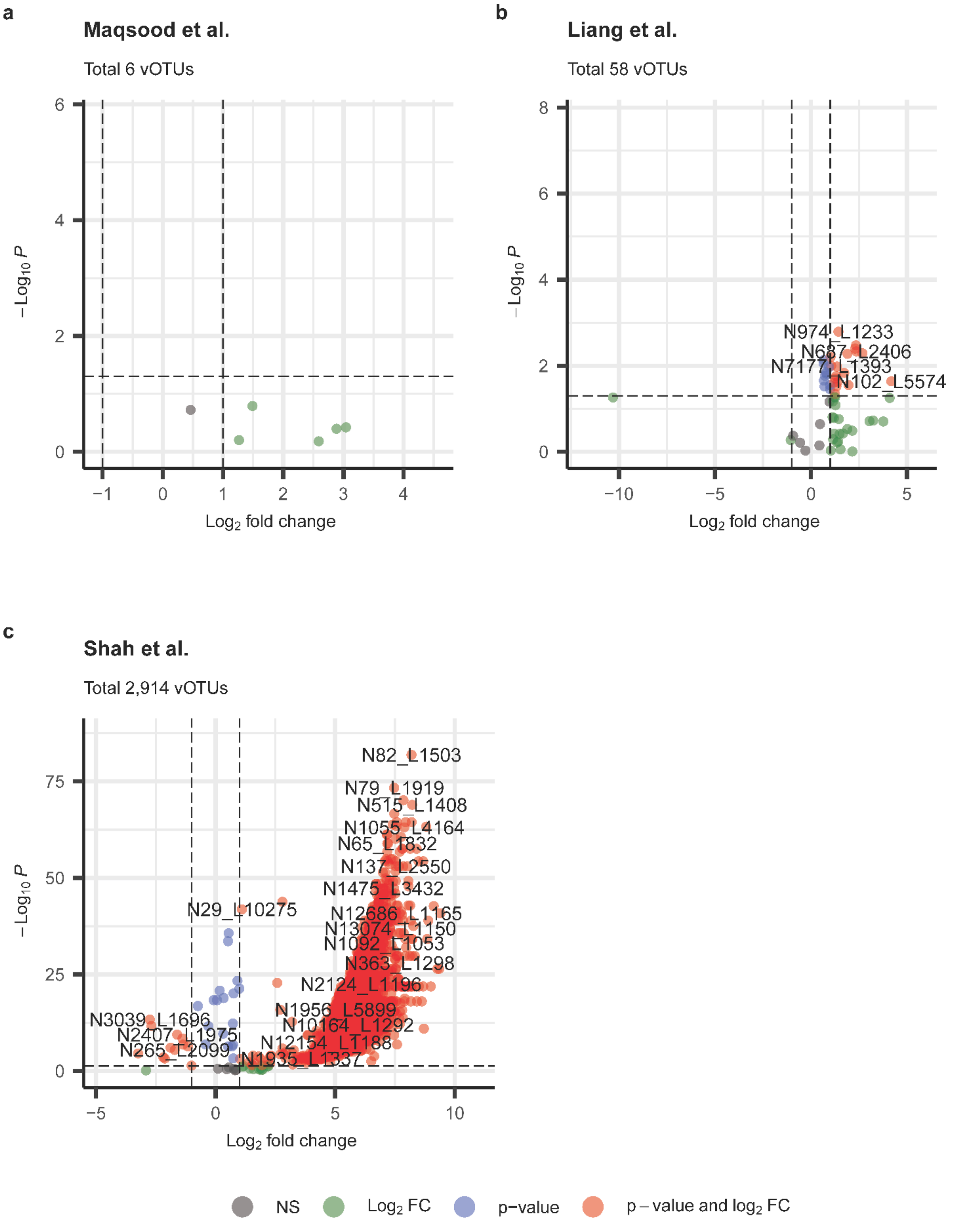

Supplementary Figure 8. Differential abundance of vOTUs shared between NCs and samples. Volcano plots for the differential abundance of vOTUs shared between NCs and samples in the studies of **a.** Maqsood et al., **b.** Liang et al., and **c.**

Shah et al. In **a-c,** every point corresponds to the vOTU detected in at least two samples and two NCs in the same study. vOTUs that were more abundant in NCs than in biological samples are shown as red dots in the upper right panels, while those enriched in samples appear as red dots in the upper left panels. Dot label represents a unique sample identifier comprised of the assembly node number and sequence length. The horizontal dashed line represents an FDR threshold of 0.05, while the vertical dashed lines denote log-fold change cut-offs of −1 and 1. Significance was calculated using linear-mixed models. Number of data points depicted: Maqsood et al. (n = 6), Liang et al. (n = 58), Shah et al. (n = 2,914). For details on the statistical test results, please see Supplementary Data 17.

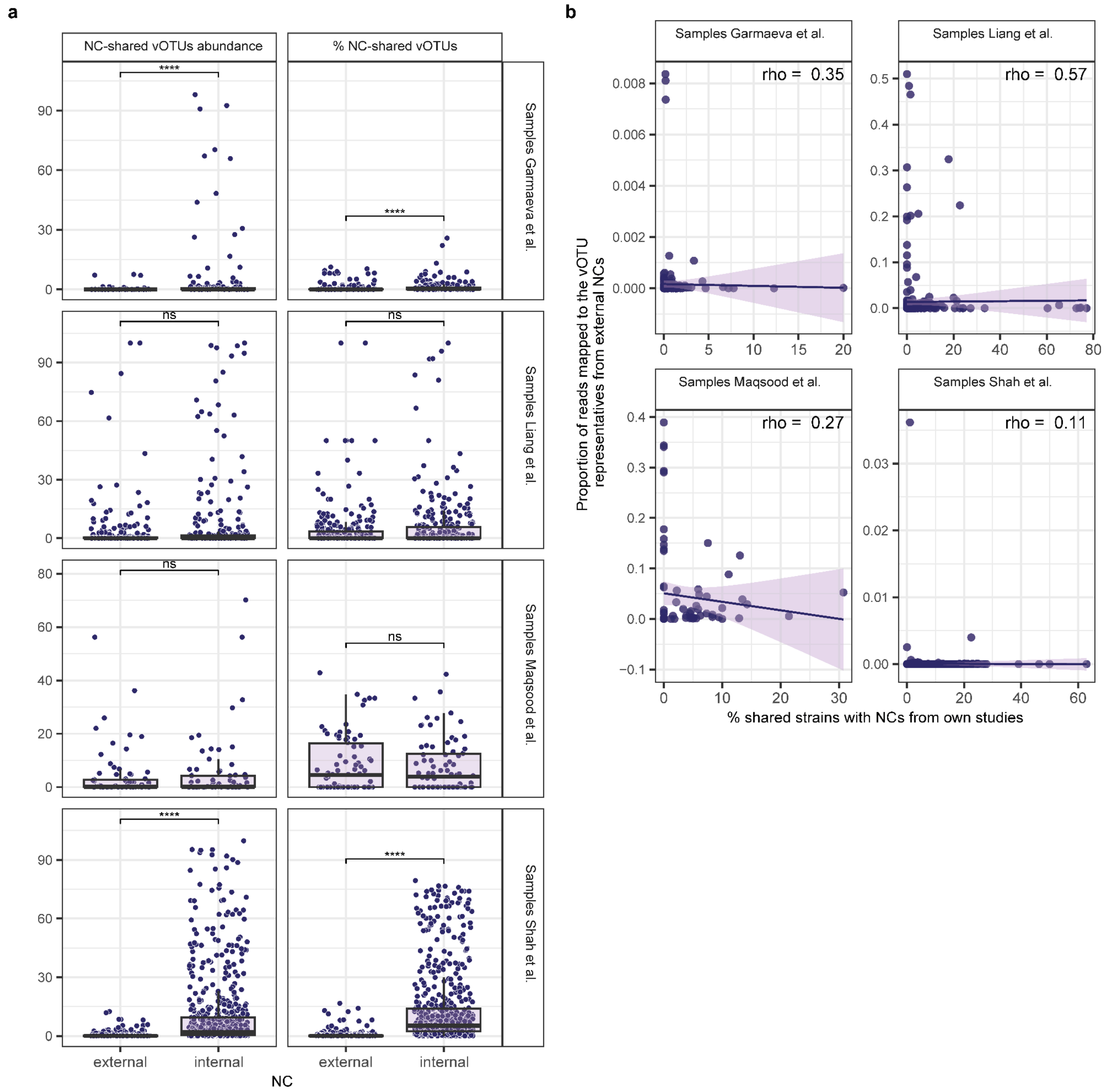

Supplementary Figure 9. vOTU and strain sharing between samples and external versus internal NCs. a. Percentage of richness and abundance represented by vOTUs shared with external versus internal NCs per study. The percentage of richness of vOTUs shared with NCs is calculated as the number of vOTUs shared with NCs divided by the total sample richness. Boxplots visualize the median, hinges (25th and 75th percentiles), and whiskers extending up to 1.5 times the interquartile range from the hinges. Asterisks denote statistical significance, ****p-value < 0.0001; ns=not significant. For details on the statistical test results, please see Supplementary Data 22. **b.** Correlation of the proportion of reads mapped to the genome sequences of vOTU representatives from external NCs with the percentage of sample richness represented by strains shared with internal NCs. The

percentage of sample richness is calculated as the number of strains shared with NCs divided by the total sample richness. The rho values depicted on the plot were obtained using the Spearman correlation test. The solid line represents the fitted linear regression, and the shaded band denotes the 95% confidence interval of the model. For details on the statistical test results, please see Supplementary Data 24. In **a-b**, data are shown for 1,254 samples. Per study: Garmaeva et al. (n = 205), Liang et al. (n = 324), Maqsood et al. (n = 78), and Shah et al. (n = 647).
