# Supplementary material for: Negativeome characterization and decontamination in early-life virome studies
Source: Nat Commun. 2025 Jul 4;16:6190. doi: 10.1038/s41467-025-61478-7 (PMC12227732; doi:10.1038/s41467-025-61478-7)
Supplement: Supplementary file 2 — Description of Additional Supplementary Files [file 41467_2025_61478_MOESM2_ESM.pdf]

Description of Additional Supplementary Files

**File Name:** Supplementary Data 1

**Description:** The file contains supporting data for the “Negativeome characterization and decontamination in early-life virome studies” publication. The first sheet, titled “Index”, provides a detailed description of the contents of the subsequent supplementary data sheets (Supplementary Data 1–25), listed in numerical order. These include cohort descriptions, results of statistical analyses performed throughout the study, sample inclusion/exclusion details for specific analyses, and comparisons of NC-detected sequences with existing databases.
